# Enteric neuro-immune-tumor ecosystem in pancreatic, colorectal, and gastric malignancies: context dependence and translational priorities

**DOI:** 10.3389/fimmu.2026.1916481

**Published:** 2026-08-20

**Authors:** Huizhong Jiang, Zijin Sun, Xinyu Lu, Kai Wang, Tinglan Cao, Sirui Wang, Linjing Song, Wei Shao, Yanju Bao, Shasha Yan, Yifei Yun

**Affiliations:** 1 Beijing University of Chinese Medicine, Beijing, China; 2 Guang’anmen Hospital, China Academy of Chinese Medical Sciences, Beijing, China; 3 Dongzhimen Hospital, Beijing University of Chinese Medicine, Beijing, China; 4 Dongfang Hospital, Beijing University of Chinese Medicine, Beijing, China

**Keywords:** context dependency, enteric nervous system, gastrointestinal malignancies, immunotherapy, neuro-immune crosstalk, perineural invasion, tumor microenvironment

## Abstract

Pancreatic ductal adenocarcinoma, colorectal cancer, and gastric cancer arise in densely innervated tissues, yet neural regulation remains incompletely integrated into tumor immunology. This narrative review uses the term “enteric neuro-immune-tumor ecosystem” for an operational framework comprising five anatomy-anchored evidence domains: local enteric nervous system remodeling, extrinsic autonomic and sensory regulation, perineural invasion-associated niches, glial-cell-mediated signaling, and microbiota-neuro-immune communication. Hepatocellular carcinoma and cholangiocarcinoma are treated as a labeled hepatobiliary extension. Evidence is described consistently as GI-direct evidence, GI-physiology evidence, or cross-tumor evidence and is interpreted separately from study maturity and translational status. Available studies indicate context-dependent associations or effects of sympathetic, parasympathetic, sensory, and glial signals on CD8+ T-cell function, myeloid-cell states, stromal organization, and cancer-cell state; the direction and magnitude vary with tumor type, disease stage, anatomical site, neural subtype, and receptor-bearing cell. Cancer-induced nerve injury is associated with IL-6- and type I interferon-related inflammation and dysfunctional antitumor immunity, whereas selected cholinergic and sympathetic circuits can restrain tumor growth in specific preclinical models. Candidate interventions, including β-adrenergic blockade, NGF/Trk inhibition, denervation, and neurotoxin-based approaches, are supported mainly by preclinical studies or retrospective associations. Prospective tumor-specific evidence and validated selection biomarkers remain limited, and none of these interventions has an approved oncologic indication for neuro-immune modulation. A context- and evidence-matched approach may help define when neural targeting merits clinical testing and when it could disrupt protective neural functions.

## 1 Introduction

### 1.1 Immune exclusion and heterogeneous immunotherapy responsiveness in gastrointestinal malignancies

Immune checkpoint inhibitors (ICIs) have transformed the management of several solid tumors, but benefit remains uneven across gastrointestinal malignancies. Poorly inflamed or immune-excluded phenotypes are common in pancreatic ductal adenocarcinoma (PDAC) and microsatellite-stable (MSS) colorectal cancer (CRC), whereas gastric cancer (GC), hepatocellular carcinoma (HCC), and cholangiocarcinoma contain molecular and etiologic subsets with distinct immune architectures and ICI responsiveness (1–3). We therefore use the term “cold tumor” only for a defined immune phenotype, not as a uniform label for an entire cancer type.

Stromal and tissue barriers restrict immune-cell access in non-equivalent ways. In PDAC, TGF-β signaling supports LRRC15+ myofibroblast states associated with CD8+ T-cell exclusion, whereas FAP+ and αSMA+ fibroblasts have distinct functions and depletion of αSMA+ cells can worsen outcomes (4, 5). Extracellular matrix and hyaluronic-acid remodeling can increase pressure and promote invasive epithelial states (6, 7). Cancer-induced nerve injury adds a spatially localized barrier linked to chronic IL-6/type I interferon signaling and reduced anti-PD-1 responsiveness (8), while SPP1+ fibro-myeloid neighborhoods may reinforce immune exclusion (9). These processes define the immune endpoints that neural inputs may modify.

Other non-neural constraints provide essential context without constituting the focus of this review. Matrix mechanosensing, obesity-associated extracellular vesicles, CXCL1-CXCR2 signaling, and YTHDF1-dependent translation can alter myeloid recruitment or differentiation (10–13); IL-35+ regulatory B cells and Fusobacterium nucleatum-derived succinate can suppress NK-cell activity or T-cell migration (14, 15). Ammonia and tumor-associated metabolic competition can constrain T-cell function (16, 18), while MED12-, lncRNA-, and CD160-associated programs affect interferon signaling and antitumor immunity (19–21). Together with emerging combination strategies (22–24), these findings show that neural regulation acts within heterogeneous stromal, metabolic, and immune states rather than creating one shared gastrointestinal tumor phenotype.

### 1.2 The unique neuroanatomy of the gastrointestinal tract: the enteric nervous system

The gastrointestinal tract contains an extensive intrinsic and extrinsic peripheral neural network. The intrinsic enteric nervous system (ENS) comprises neurons within the myenteric and submucosal plexuses, including choline acetyltransferase- and nitric oxide synthase-immunoreactive populations (25, 26), together with enteric glia. Schwann cells ensheath extrinsic peripheral axons, whereas enteric glia are interwoven with intrinsic neurons. These cellular populations contribute to motility, blood flow, nutrient handling, epithelial-barrier integrity, and immune regulation (27, 28). Their close apposition to epithelial, stromal, and immune compartments creates multiple potential sites of neural interaction in gastrointestinal disease.

Extrinsic innervation includes vagal, sympathetic, and pelvic pathways. The vagus nerve supplies much of the upper gastrointestinal tract and participates in gastric efferent activity and emetic reflexes (25, 29, 30). In the pelvis and rectum, the superior hypogastric plexus, hypogastric nerves, pelvic plexus, and associated neurovascular bundles occupy defined fascial planes (17, 31). High-resolution imaging and cadaveric studies further define the three-dimensional topology of these plexuses, whose preservation is relevant to postoperative organ function (32).

This dual intrinsic-extrinsic organization provides an anatomical basis for neuro-immune-tumor interactions, but proximity alone does not establish functional regulation. Gastric tumors can alter the distribution of cocaine- and amphetamine-regulated transcript (CART)- or galanin-containing fibers (33), whereas oxaliplatin can reduce myenteric neurons and change glial density, contributing to treatment-related gastrointestinal dysfunction (25, 26). Enteric glia and vagal endings can also influence inflammatory signaling through neurotransmitters and neuropeptides (27, 28). These observations identify candidate interfaces that require tumor-, cell-, and receptor-specific validation.

### 1.3 Conceptual scope and dual-axis evidence classification

**Figure 1:**
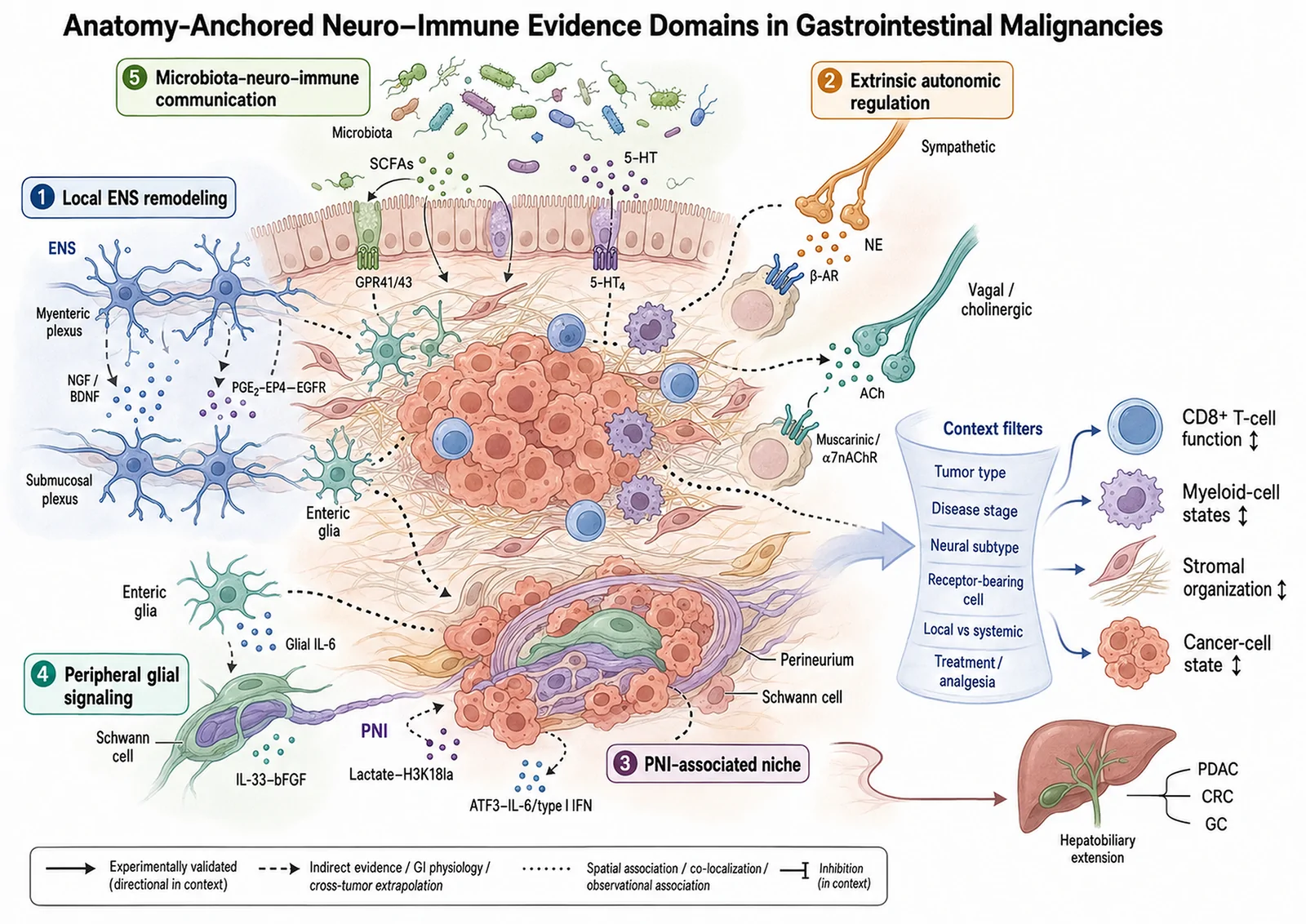
Anatomy-anchored neuro-immune evidence domains in gastrointestinal malignancies. Five domains—local ENS remodeling, extrinsic autonomic and sensory regulation, the PNI-associated niche, peripheral glial signaling, and microbiota-neuro-immune communication—are interpreted through tumor type, disease stage, neural subtype, receptor-bearing cell, local versus systemic signaling, and treatment or analgesia. Outputs are context dependent. Solid arrows indicate experimentally supported direction in the stated context; dashed arrows indicate indirect evidence, GI-physiology evidence, cross-tumor evidence, or incomplete links; dotted lines indicate spatial or observational associations. PDAC, CRC, and GC constitute the core scope; HCC and CCA form the hepatobiliary extension.

We use “enteric neuro-immune-tumor ecosystem” as a singular operational framework comprising five anatomy-anchored evidence domains: (i) local remodeling of enteric neurons and plexuses; (ii) extrinsic sympathetic, parasympathetic, and sensory regulation; (iii) perineural invasion (PNI)-associated niches; (iv) enteric glial- and Schwann-cell-mediated signaling; and (v) microbiota-neuro-immune communication. These domains are not mutually exclusive: local ENS remodeling and glial signaling intersect at enteric glia, while the microbiota domain spans the gut wall and inter-organ neural circuits. They serve as a mapping framework for distinct anatomical contexts and convergent immune outputs rather than as five fully separate compartments (**Table 1**; **Figure 1**). Evidence provenance captures disease-context relevance: GI-direct evidence is generated in a gastrointestinal or hepatobiliary malignancy; GI-physiology evidence derives from non-malignant gastrointestinal or hepatobiliary physiology or inflammation; and cross-tumor evidence is extrapolated from a non-gastrointestinal malignancy. Evidence maturity and translational status are recorded separately as preclinical (*in vitro*, ex vivo, or animal), retrospective human, prospective human, established supportive or non-oncologic clinical use, or approved oncologic use. Provenance does not indicate methodological quality, and maturity or status does not replace a formal risk-of-bias assessment. PDAC, CRC, and GC form the core scope; HCC and cholangiocarcinoma are included only as a clearly labeled hepatobiliary extension. Across all domains, conclusions are bounded by tumor type, disease stage, anatomical site, neural subtype, receptor-bearing cell, and local versus systemic signaling.

**Table 1:** Operational, anatomy-anchored evidence domains in the enteric neuro-immune-tumor ecosystem.

Evidence domain	Anatomy/components	Representative axes and readouts	Provenance, maturity, and status	Interpretive boundary
Local ENS remodeling	Myenteric/submucosal plexuses; enteric neurons and glia	NGF/BDNF, PGE2-EP4-EGFR, IL-1/IL-6; neural density and glial-state changes	GI-direct evidence and GI-physiology evidence; predominantly preclinical, with retrospective human associations; stage-dependent glial loss and re-emergence (56, 99, 101, 102, 141–144)	Causality and prognostic utility require prospective spatial validation.
Extrinsic autonomic regulation	Sympathetic, vagal, pelvic, and splanchnic inputs	NE-β-AR-cAMP/PKA; ACh-muscarinic or α7nAChR signaling	Mixed GI-direct evidence, GI-physiology evidence, and cross-tumor evidence; predominantly preclinical, with limited retrospective human evidence (78–89, 127, 136–140, 149)	Direction varies by nerve subtype, receptor-bearing cell, local versus systemic signaling, and disease stage.
PNI-associated niche	Perineurium, invaded nerves, fibroblasts, Schwann cells, and myeloid cells	ACh-HDAC1-CCL5; CXCL12-CXCR4/CXCR7; lactate-H3K18la; CINI-ATF3-IL-6/type I IFN	GI-direct evidence and cross-tumor evidence; predominantly preclinical, with retrospective human associations; strongest in PDAC and GC (8, 62–68, 127–133)	PNI is a context-dependent spatial niche, not intrinsically immune privileged in all tumors.
Peripheral glial signaling	Enteric glia, Schwann cells, and other tissue-resident glia	PGE2-EP4-EGFR; glial IL-6; Schwann-cell tracks; IL-33-bFGF loops	Mixed GI-direct evidence, GI-physiology evidence, and cross-tumor evidence; predominantly preclinical, with retrospective human associations (99–105, 130–133)	Homeostatic glial functions create safety concerns for systemic inhibition.
Microbiota-neuro-immune communication	Microbial metabolites, epithelium, ENS, vagal afferents, and splenic circuits	SCFA-GPR41/43; 5-HT-5-HT4; splenic NE-β2-AR-ACh-α7nAChR reflex	GI-physiology evidence; predominantly preclinical; tumor-specific circuits remain incomplete (106–116, 119, 122–125)	Do not infer a complete microbiota-vagus-tumor causal chain from separate component studies.

Evidence provenance: GI-direct evidence, generated in a gastrointestinal/hepatobiliary malignancy; GI-physiology evidence, derived from non-malignant gastrointestinal/hepatobiliary physiology or inflammation; cross-tumor evidence, extrapolated from a non-gastrointestinal malignancy. Evidence maturity and translational status: preclinical, retrospective human, prospective human, established supportive/non-oncologic clinical use, or approved oncologic use. These axes are descriptive and do not constitute a formal risk-of-bias assessment.

Recent reviews have synthesized peripheral-nerve biology, sensory neurotransmission and pain, neuro-immune signaling, therapeutic targets, enabling technologies, and trial design across tumor types (34–43). Among the closest gastrointestinal-focused comparisons, Chen and Tang integrate ENS-, immune-, and microbiota-mediated regulation across gastrointestinal tumors and emphasize spatiotemporal and translational themes, including PNI, Schwann cells, and enteric glia (44), whereas Zhao et al. organize immune-neural mechanisms around chronic inflammation, molecular pathways, gastric and colorectal cancer, other gastrointestinal malignancies, and therapeutic innovation, while also discussing PNI, microbiota, disease stage, and clinical translation (45). The present review is therefore not distinguished simply by including the ENS, PNI, glia, or microbiota. Its added value is an evidence-mapping structure that defines PDAC, CRC, and GC as the core disease scope, treats HCC and cholangiocarcinoma as a labeled hepatobiliary extension, maps findings onto five operational, anatomy-anchored domains, and interprets mechanistic and clinical inferences by evidence provenance, evidence maturity and translational status, disease stage, receptor-bearing cell type, and anatomical context. This structure makes explicit which conclusions are supported by GI-direct evidence and which remain GI-physiology evidence or cross-tumor evidence.

### 1.4 Literature search and evidence curation

This narrative review was informed by iterative searches of PubMed/MEDLINE and Google Scholar for English-language literature published from January 2015 through July 2026, supplemented by backward and forward citation tracking of key primary studies and recent reviews. Search terms were combined around five themes: (i) enteric nervous system, autonomic or vagal signaling, sympathetic signaling, sensory neurons, and neuropeptides; (ii) PNI, cancer-induced nerve injury, Schwann cells, and enteric glia; (iii) microbiota-neuro-immune, gut-brain, and splenic pathways; (iv) PDAC, CRC, GC, HCC, and cholangiocarcinoma; and (v) CD8+ T cells, myeloid cells, stromal organization, immune checkpoint therapy, and neural interventions. Peer-reviewed primary studies were prioritized over reviews for mechanistic claims. Evidence was then mapped in the order GI-direct evidence, GI-physiology evidence, and cross-tumor evidence, with prospective human studies prioritized over retrospective associations and preclinical models when addressing translational questions. Preprints, conference abstracts, unpublished data, and other non-peer-reviewed sources were excluded. Because this is a narrative rather than systematic review, no formal protocol registration, duplicate screening, meta-analysis, or study-level risk-of-bias assessment was undertaken.

Across these domains, ligand-receptor context determines the immune outcome. Norepinephrine (NE)-β2-adrenergic receptor (β2-AR)-cAMP/PKA signaling can reduce CD8+ T-cell metabolic fitness and cytokine production, whereas acetylcholine (ACh) can signal through the α7 nicotinic acetylcholine receptor (α7nAChR) or muscarinic receptors on macrophages, lymphocytes, stromal cells, and tumor cells. Relevant readouts include CD8+ T-cell infiltration, IFN-γ and granzyme production, inhibitory-receptor expression, dendritic-cell activation, NK-cell cytotoxicity, chemokine gradients, and the spatial distribution of monocyte-derived, tissue-resident, and SPP1+ macrophage states (36, 46–49). These axes should therefore be interpreted at the level of receptor-bearing cell type and anatomical context rather than by neurotransmitter identity alone.

Sensory-neuron regulation provides a useful example of evidence boundaries. In a preclinical head and neck squamous cell carcinoma model, an ATF4-SLIT2 circuit was reported to activate nociceptors and induce calcitonin gene-related peptide (CGRP) release, with downstream changes in CCL5 and macrophage states in tumor-draining lymph nodes (50). For gastrointestinal oncology, this finding constitutes cross-tumor evidence with preclinical maturity: it supports a biologically plausible circuit but does not demonstrate the same pathway in PDAC, CRC, GC, HCC, or cholangiocarcinoma. Systemic or axonal-reflex propagation is likewise supported in broader neuroimmunology (51, 52) but remains incompletely mapped in gastrointestinal tumors.

Selected gastrointestinal studies provide more direct evidence for glial regulation. In CRC, IL-1R-dependent signaling from infiltrating monocytes can alter enteric glial states, while glial IL-6 can support differentiation toward SPP1+ TAM programs (53). Tumor-associated Schwann cells can also undergo injury-associated reprogramming and influence inflammatory and stromal organization in specific models (54). Reports of checkpoint-molecule expression on nerve fibers (43) are hypothesis-generating and require cell-type-resolved validation. Collectively, these data support further testing of neural targets but do not establish uniform benefit from β-blockers, CGRP antagonists, or other neuromodulators.

## 2 Anatomical remodeling: tumor neurogenesis and the construction of physical barriers

### 2.1 Tumor-induced neoneurogenesis

Gastrointestinal tumor and stromal cells can secrete neurotrophic factors that promote pathological axonal growth toward the tumor (neoneurogenesis) (55). NGF and brain-derived neurotrophic factor (BDNF) provide chemotactic and trophic signals, but their contribution varies by tumor and experimental setting. In PDAC, serine/glycine deprivation causes ribosomal stalling and selective NGF translation, and the resulting NGF supports tumor innervation in nutrient-poor conditions (56). HGF/c-Met-mTOR signaling can also increase NGF and perineural invasion in pancreatic models (57).

Experimental models support reciprocal signaling between nerves, tumor cells, and fibroblasts. In PDAC and CRC, sympathetic NE can act through β2-AR on tumor cells or cancer-associated fibroblasts to increase NGF, and fibroblast-derived NGF can in turn increase sympathetic-fiber density (58, 59). In an intrahepatic cholangiocarcinoma model, ACh-α5 nicotinic receptor signaling was associated with increased BDNF and neural infiltration (60). In PDAC, nociceptor-fibroblast communication through NGF and CGRP can influence axonal growth and NK-cell recruitment or function (61). Tumor-derived extracellular vesicles provide an additional candidate mechanism for long-range axonal guidance (55). These data support model-specific feedforward circuits rather than a single universal neurotrophic program.

### 2.2 Perineural invasion-associated immune niches

PNI has traditionally been viewed as tumor spread along nerve sheaths (62). Multi-omic and spatial studies now support a broader model in which the perineural compartment can acquire immune-regulatory features, although the strength of evidence varies by tumor type (63). PNI-associated cancer-associated fibroblasts can undergo glycolytic reprogramming, and lactate uptake by tumor cells can promote H3K18 lactylation and expression of neural-invasion genes such as L1CAM and SLIT1 (64). Because lactate can also impair effector lymphocyte function, this mechanism links metabolic remodeling to a plausible, rather than universally established, route of local immune suppression.

The PNI compartment may also alter cytokine and chemokine gradients. In pancreatic and non-gastrointestinal cancers, tumor-derived extracellular vesicles carrying regulatory RNAs can induce macrophage states characterized by CD206, ARG1, or IL-10, and these cells can facilitate neural invasion (63, 65–67). We retain the marker terminology reported by the source studies but avoid treating these cells as a single stable “M2” lineage. Axon-guidance pathways such as SEMA3D-PLXND1 can promote directed migration (68); their contribution to immune exclusion in each gastrointestinal cancer remains to be established. .

**Figure 2:**
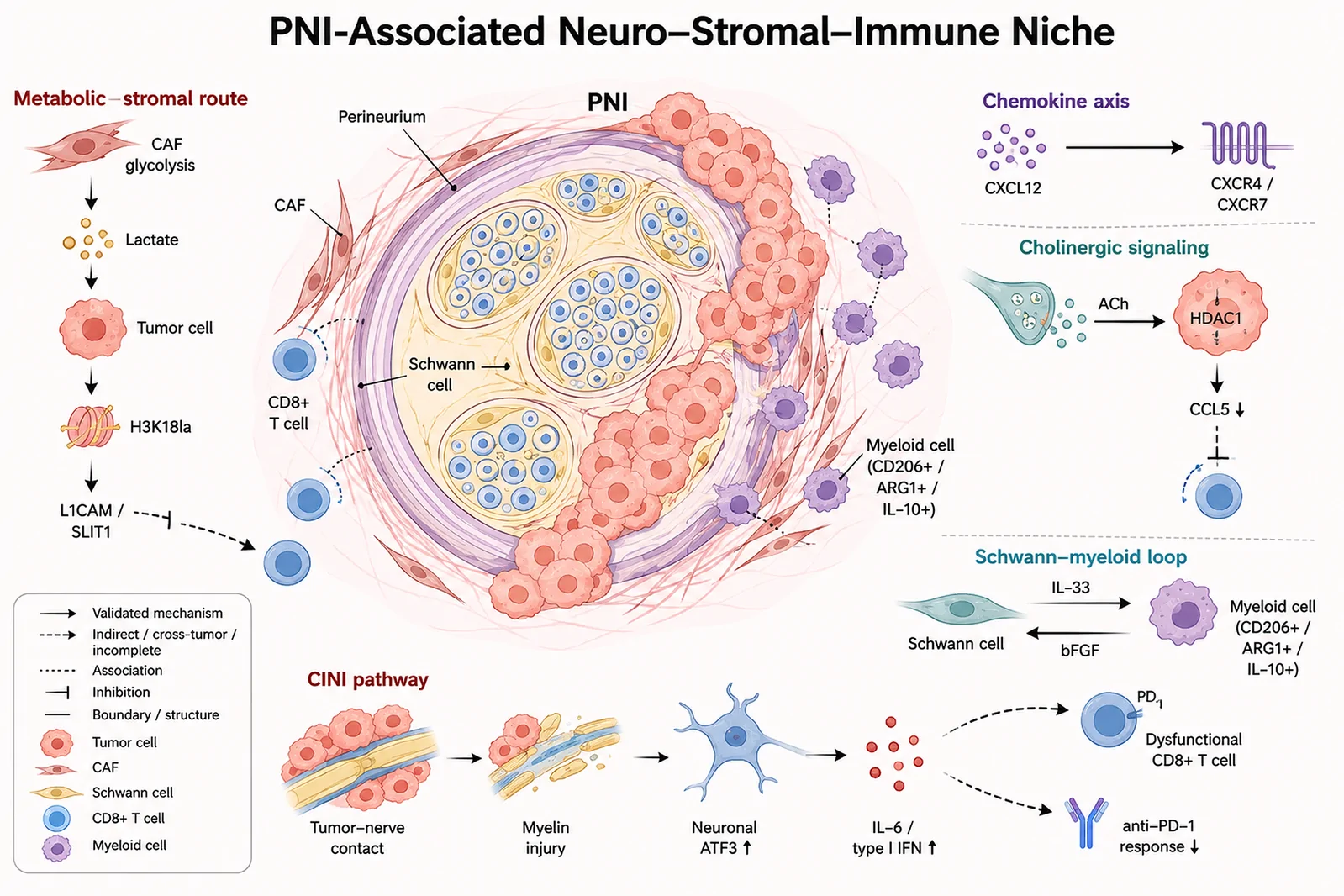
PNI-associated neuro-stromal-immune niche. The schematic integrates CAF-derived lactate-H3K18la signaling, CXCL12-CXCR4/CXCR7, ACh-HDAC1-CCL5 regulation, a Schwann-cell-IL-33/myeloid-bFGF loop, and cancer-induced nerve injury associated with neuronal ATF3, IL-6/type I IFN, CD8+ T-cell dysfunction, and reduced anti-PD-1 response. These routes derive from selected models and should not be interpreted as a universal immune-privileged niche.

Accordingly, PNI is best described as a spatially restricted neuro-stromal-immune niche rather than an intrinsically immune-privileged sanctuary. The perineurium, fibroblasts, Schwann cells, metabolites, and recruited myeloid cells can collectively limit cytotoxic-cell access in some models, but causal evidence is strongest in PDAC and remains incomplete in other gastrointestinal malignancies (62–65) (**Figure 2**).

Cancer-induced nerve injury (CINI) provides a model-specific mechanistic link between PNI and reduced immunotherapy responsiveness. In GC and other neurotropic cancers, tumor-cell contact can degrade myelin and activate neuronal ATF3, followed by IL-6- and type I interferon-associated inflammatory signaling. As injury accumulates, this repair-oriented response can become chronic and is associated with suppressive and exhaustion-related immune states. In experimental systems, Ifnar1 loss, neuronal Atf3 deletion, denervation, or combined IL-6-receptor and PD-1 blockade improved tumor control (8). The clinical association includes GC, whereas several intervention experiments were performed in non-gastrointestinal models; we therefore distinguish the human association from the preclinical causal tests.

### 2.3 Neuro-immune interfaces

Neural regulation in tumors can occur through volume transmission, receptor-specific paracrine signaling, and—in selected tissues—close contacts between nerve endings and immune cells. We use “neuro-immune interface” rather than assuming that every interaction is a canonical synapse (69, 70). Tumor-draining lymph nodes are innervated and can integrate sensory or sympathetic signals, but direct millisecond-scale control has not been established for most gastrointestinal tumor-immune pairs. Observations of cholinergic fibers near CD133+ cells in thyroid cancer and selected gastrointestinal models (71) are therefore interpreted as spatial association unless functional receptor dependence was experimentally demonstrated.

Several mechanistic examples in this area derive from non-gastrointestinal models and therefore constitute cross-tumor evidence with predominantly preclinical maturity. In triple-negative breast cancer, β-adrenergic signaling in perivascular macrophages can promote VEGF/CD206-associated states and reduce T-cell activation (72). In bone-cancer pain models, substance P (SP)-neurokinin-1 receptor (NK-1R) signaling recruits macrophages (73), while tumor-derived small extracellular vesicles can sensitize nociceptors and promote SP/IL-6-associated CD8+ T-cell dysfunction in head and neck cancer and melanoma (74). These findings support testing β2-AR, CCR2, or NK-1R interventions in gastrointestinal models (36, 69, 70, 75, 76), but they should not be presented as direct evidence of efficacy in gastrointestinal malignancies.

## 3 Molecular mechanisms: neurotransmitter reprogramming of the immune microenvironment

### 3.1 Sympathetic signaling and CD8+ T-cell dysfunction

**Figure 3:**
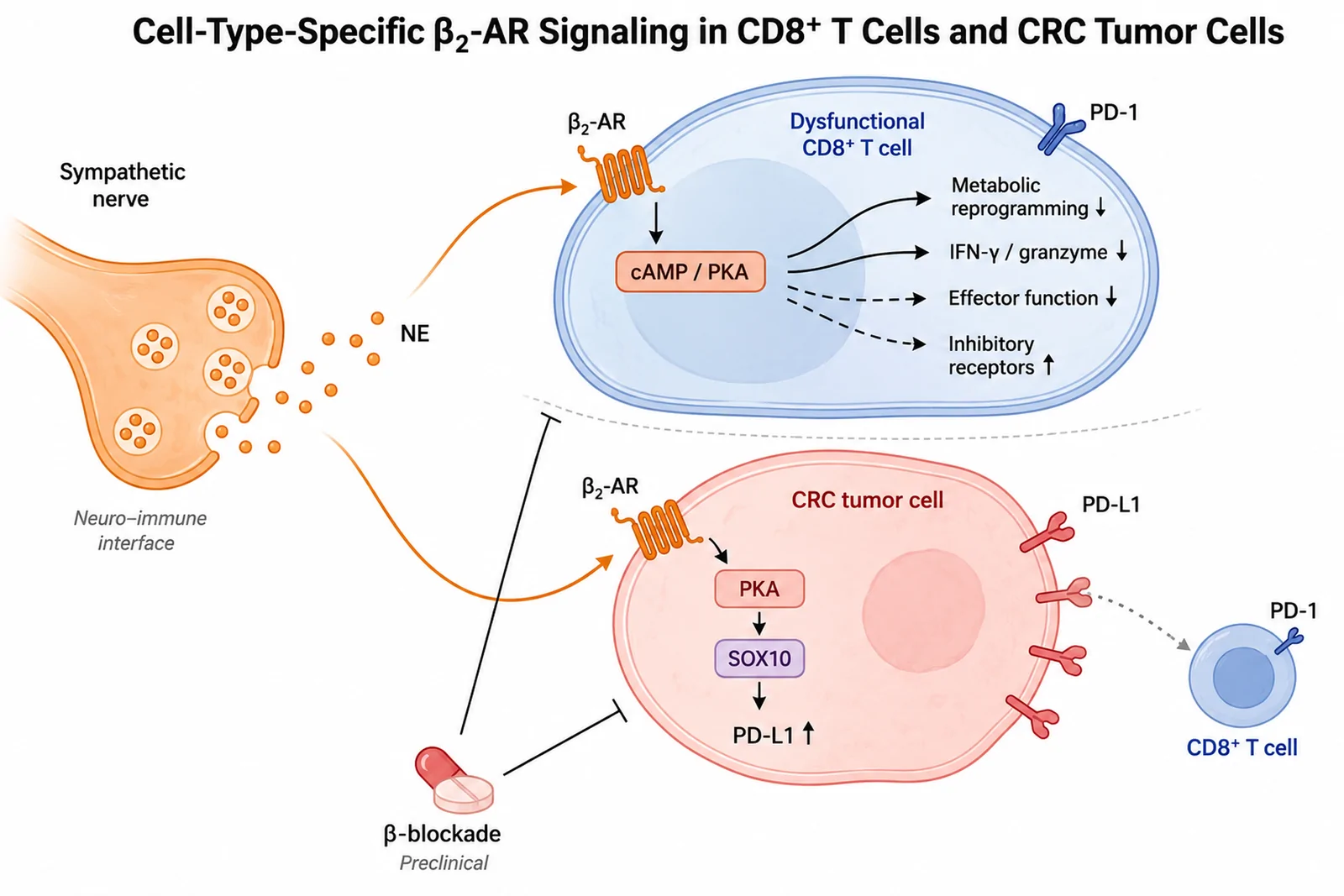
Cell-type-specific β2-adrenergic signaling in CD8+ T cells and CRC tumor cells. Sympathetic NE-β2-AR-cAMP/PKA signaling can impair CD8+ T-cell metabolic reprogramming, IFN-γ/granzyme production, and effector function in preclinical models. In CRC cells, β2-AR-PKA-SOX10 signaling can increase PD-L1. β-blockade is shown as a preclinical strategy; the two cell-specific arms should not be assumed to have identical evidence provenance or clinical maturity.

Chronic sympathetic activation can suppress antitumor immunity through NE-β2-AR signaling in defined experimental contexts. In stress models, β2-AR engagement on CD8+ T cells impairs metabolic reprogramming, cytokine production, and effector function (77). In PDAC and other tumors, dysfunctional CD8+ T cells have been observed near sympathetic fibers, and β-adrenergic blockade can favor tissue-resident-memory-like programs in preclinical settings (78) (**Figure 3**). A β2-AR-PKA-SOX10-PD-L1 axis has also been reported in CRC and melanoma tumor cells (79). The CRC findings are GI-direct evidence, whereas the melanoma findings are cross-tumor evidence.

In the MC38 colon cancer model, propranolol reduced angiogenesis and MDSC abundance, increased effector T-cell infiltration, and improved anti-CTLA-4 activity (80). Chronic stress can also activate ADRB2 signaling in GC and impair CD8+ T-cell function, with interaction between stress hormones and Helicobacter pylori-associated inflammation (81). These are preclinical findings. Human survival associations with β-blocker exposure remain heterogeneous, particularly in pancreatic cancer (82), and do not establish treatment efficacy.

### 3.2 Parasympathetic and cholinergic signaling: context-dependent immune effects

Parasympathetic and non-neuronal cholinergic signals regulate gastrointestinal immunity through receptor- and context-dependent mechanisms. Under physiological or acute inflammatory conditions, ACh can engage α7nAChR on macrophages, reduce NF-κB nuclear translocation, and activate JAK2/STAT3-associated responses, with lower TNF-α, IL-6, and IL-1β production (83, 84). Acute liver-injury, metabolic-liver-disease, and inflammatory-bowel-disease models also report protection from excessive inflammation or tissue injury (84–86). These non-tumor observations establish physiological plausibility but do not by themselves predict the direction of cholinergic signaling in an established malignancy.

In tumor and chronic inflammatory contexts, α7nAChR signaling can support macrophage states characterized by CD206, ARG1, reduced inflammatory cytokine production, or impaired antigen-presenting function (46, 87, 88). These marker-defined states are often described as “M2” in the source literature, but their ontogeny—monocyte-derived versus tissue-resident—and their spatial relationship to tumor cells are rarely resolved. Cholinergic regulation of postoperative ileus (89) constitutes GI-physiology evidence, with both preclinical mechanistic and prospective clinical support, and should not be interpreted as proof that the same circuit causes tumor immune escape.

Hepatobiliary examples further illustrate context dependence. Nicotine or cholinergic agonists can activate α7nAChR-JAK2-STAT3 and PI3K-AKT signaling in HCC models (90). Separately, vagal regulation of CXCL9 has been studied in severe liver inflammation (91); this constitutes GI-physiology evidence with clinical association and preclinical causal testing and supports a testable hypothesis about effector-cell recruitment without establishing tumor-specific suppression. Thus, cholinergic stimulation may protect tissue during acute inflammation yet have different consequences in an established tumor.

### 3.3 Sensory neurons and neuropeptides in chronic inflammation

Sensory neurons can sustain neurogenic inflammation through SP, CGRP, and other mediators, but GI-direct evidence remains limited. Tumor-derived extracellular vesicles can increase sensory-neuron growth and SP/IL-6 release in non-gastrointestinal tumor models (74), while mast-cell degranulation and neuronal nitric oxide synthase participate in pain-associated feedback circuits (92). Chemotherapy-induced neuropathy models additionally show macrophage and mast-cell recruitment with TNF-α and IL-1β production (93, 94). This cross-tumor evidence and treatment-toxicity evidence are predominantly preclinical and inform mechanistic hypotheses, but they should not be equated with spontaneous gastrointestinal tumor immunity.

NK-1R and CGRP pathways remain candidate intervention points. NK-1R blockade can inhibit proliferation or induce apoptosis in several tumor models (95, 96), and chemogenetic activation of TRPV1+ sensory neurons can alter macrophage and MDSC states (97). Because sensory circuits can also support immune surveillance in other models, prospective gastrointestinal studies should define nerve subtype, receptor-bearing immune population, disease stage, and analgesic exposure before therapeutic inhibition is considered.

### 3.4 Enteric glial cells and schwann cells

In CRC, tumor-derived signals can induce reactive enteric glial states that differ from homeostatic barrier-supporting glia (98, 99). Enteric glial gene-expression signatures have been associated with prognosis and immune infiltration (100), but such retrospective signatures do not by themselves establish that glial activity causes treatment resistance.

Mechanistic studies support paracrine tumor-glial signaling. Tumor-activated enteric glia can increase PGE2 synthesis; PGE2-EP4 signaling in CRC stem-like cells activates EGFR and supports tumorigenicity (101). In colonic tumor models, c-Jun-dependent glial IL-6 can promote tumor growth (102). These pathways define specific ligand-receptor axes and functional readouts, rather than a generalized tumor-promoting glial phenotype.

Pharmacological inhibition of glial PGE2 synthesis, interference with tumor-derived IL-1, or glial c-Jun deletion reduced tumor-supporting phenotypes in preclinical models (101, 102). These results identify PGE2-EP4-EGFR and IL-1/IL-6 pathways as experimental targets; safety and selectivity remain important because enteric glia maintain epithelial, neuronal, and barrier homeostasis.

Peripheral glia beyond enteric glial cells also shape cancer-neural interfaces. In PDAC, cancer-activated Schwann cells can form migratory tracks that facilitate invasion (103), and reviews of peripheral-cancer Schwann-cell biology emphasize injury-like plasticity, axon guidance, extracellular-matrix remodeling, and immune signaling (104). Tissue-resident glial cells have also been associated with tumor vasculature and cancer progression in non-gastrointestinal models (105). The first observation is GI-direct evidence; the broader vascular-glial mechanism is cross-tumor evidence supported by preclinical experiments and retrospective human data.

## 4 Microbiota-neuro-immune communication

This section examines how short-chain fatty acids (SCFAs) and 5-HT influence ENS excitability, neuro-immune signaling, and gastrointestinal tumor biology.

### 4.1 Microbial metabolites as neuromodulators

Microbial metabolites influence the development and function of the ENS, but cancer-specific causal chains are incompletely resolved. Short-chain fatty acids (SCFAs) signal through receptors including GPR41 and GPR43 on neural, glial, epithelial, and immune populations (106, 107). Butyrate can inhibit histone deacetylases and support enteric-neuron resilience in inflammatory models (107, 108), while microbiota depletion and restoration experiments implicate SCFAs in enteric neurogenesis (109). Effects on IL-22, barrier integrity, and myeloid differentiation (110, 111) have GI-physiology evidence and are supported predominantly by preclinical evidence for gastrointestinal oncology. Microbiota-associated serotonin (5-HT) can regulate 5-HT4-dependent enteric-neuron maturation and epithelial proliferation (112–115), but whether these effects promote or restrain CRC depends on inflammatory and receptor context (116).

These observations support a microbiota-metabolite-ENS framework in which SCFAs, 5-HT, GABA, dopamine, and neurotrophic signals can alter neural excitability and local immune tone (117–119). However, increasing SCFAs or manipulating 5-HT cannot yet be considered an established anticancer strategy. Interventions should be tested with tumor-specific endpoints, defined microbial communities, and simultaneous measurement of neural and immune compartments (106, 120).

### 4.2 The microbiota-gut-brain-immune axis

Gut microbial signals can reach the central nervous system through vagal afferents and can influence peripheral immunity through autonomic and splenic circuits (121, 122). In lipopolysaccharide and sleep-deprivation models, vagotomy or splenectomy modifies IL-6 and TNF-α responses (122, 123). These are preclinical, non-tumor experiments classified as GI-physiology evidence; they establish physiological plausibility but not a tumor-specific microbiota-vagus mechanism.

The splenic inflammatory reflex includes NE release from splenic nerves, β2-AR signaling in CD4+ T cells, ACh production, and α7nAChR-dependent suppression of TNF in myeloid cells (124, 125). In cancer, the direction of this pathway cannot be inferred from inflammatory models alone. Published evidence currently supports a context-dependent hypothesis: reflex signaling may limit damaging inflammation, but chronic or tumor-co-opted signaling could also reduce effective immune surveillance.

**Figure 4:**
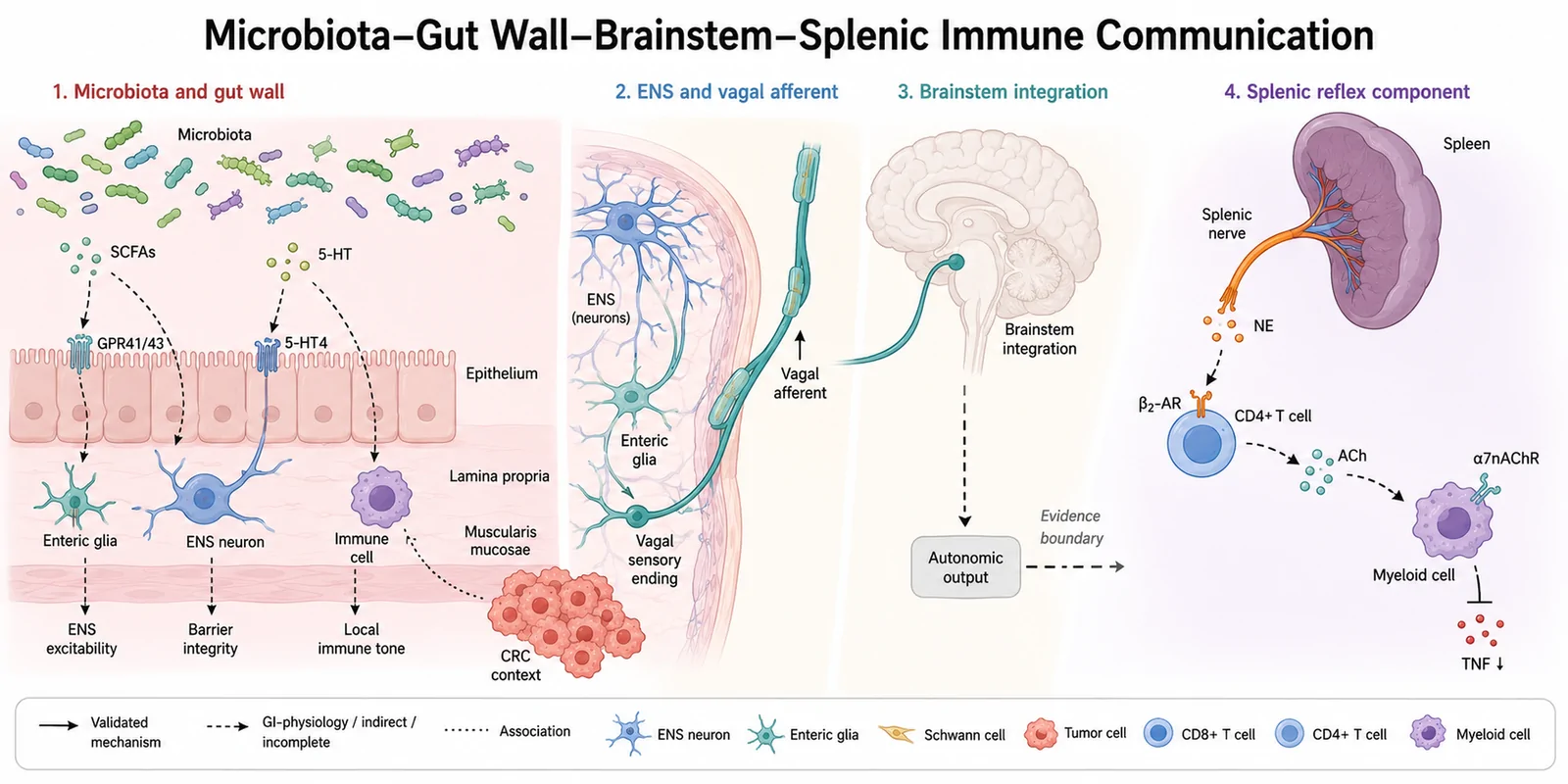
Microbiota-gut wall-brainstem-splenic immune communication. The figure separates microbial SCFA/5-HT signaling in the gut wall, ENS and vagal-afferent sensing, brainstem/autonomic integration, and the splenic NE-β2-AR-CD4+ T-cell-ACh-α7nAChR-myeloid-cell reflex. The evidence boundary emphasizes that component mechanisms are supported mainly by GI-physiology evidence and preclinical studies, whereas a complete causal microbiota-vagus-spleen-CRC circuit has not been established in patients.

CRC and other intestinal tumors can alter microbiota composition, motility, neural activity, and local immunity (117, 126). Nevertheless, a complete causal sequence from dysbiosis to vagal output to tumor immune exclusion has not yet been demonstrated in patients. We therefore present microbiota-neural modulation as an emerging research direction, with the strongest current support arising from separate microbial, neural, and immune experiments rather than a single validated circuit (**Figure 4**).

## 5 Neuro-immune landscapes in specific disease contexts

### 5.1 Pancreatic ductal adenocarcinoma: neural remodeling in an immune-excluded microenvironment

PDAC commonly combines extensive PNI with low effector T-cell infiltration. This spatial association supports, but does not by itself prove, a causal relationship between neural density and immune exclusion. Mechanistic studies now identify several local circuits through which nerves, Schwann cells, fibroblasts, and immune cells may interact.

In PDAC specimens and models, greater neural involvement has been associated with fewer CD8+ and Th1 cells and a higher Th2/Th1 balance (127). In a reported PDAC model, PNI-associated ACh reduced tumor-cell CCL5 through HDAC1 and was associated with lower CD8+ T-cell IFN-γ production (127). CXCL12-CXCR4/CXCR7 signaling is also associated with PNI, T-cell exclusion, and outcome (128, 129). These findings define measurable ligand-receptor axes and spatial readouts rather than a unitary “neural dominance” state.

Schwann cells and fibroblasts provide a cellular bridge between neural injury and myeloid remodeling. PDAC-derived extracellular vesicles and TGF-β can induce Schwann-cell dedifferentiation (130, 131). Schwann-cell IL-33 and macrophage-derived basic fibroblast growth factor (bFGF) participate in reciprocal activation, with enrichment of CD206/ARG1-associated TAM states in some models (132, 133). Nociceptor-CAF signaling through CGRP and NGF can reduce CAF IL-15 and weaken NK-cell recruitment or cytotoxicity (61).

Experimental interruption of these circuits can alter tumor growth or immunity, but the direction depends on the manipulated nerve subtype and timing. In one PDAC model, vagotomy reduced PNI-associated cholinergic signaling and increased CD8+ T-cell infiltration (127). Pharmacological disruption of SLC1A5/SLC7A5-NR2A metabolic coupling or local lidocaine delivery also reduced neural interactions in preclinical systems (134, 135). These findings do not establish that denervation will uniformly improve immunotherapy in patients.

### 5.2 Gastric cancer: context-dependent vagal and cholinergic effects

Vagal and cholinergic signals influence GC-cell proliferation, stem-like states, metabolism, and immunity, but their net effect varies by receptor, anatomical site, and disease stage. ACh can promote diffuse-type GC sphere formation and stem-cell markers (136), while CHRM3 accumulation after PJA2 loss activates TGF-β-SMAD3 signaling (137). These tumor-cell-autonomous effects should be distinguished from vagal regulation of immune cells and metastatic niches (138).

Vagal manipulation can also alter GC metabolism. In mouse models, vagotomy shifted tumor-cell metabolism from a glutamine-dependent state toward oxidative phosphorylation/glycolysis and reduced tumor growth; SNAP25, mTOR, and PDP1/α-KGDH were implicated (139). Intratumoral botulinum toxin A and metabolic combinations are therefore investigational hypotheses rather than established anticancer treatments.

Vagal effects can differ between primary and metastatic settings. In a mouse model of peritoneal metastasis, subdiaphragmatic vagotomy increased the number and grade of metastatic nodules and altered F4/80+ macrophage and CD3+ T-cell distributions within omental milky spots (140). These data suggest a protective function of intact vagal signaling in that model, but they do not establish a universal antimetastatic pathway. Any clinical evaluation of vagotomy must therefore distinguish the targeted nerve, disease site and stage, and symptom-control indication.

### 5.3 Colorectal cancer: stage-dependent neural plasticity

Across CRC development, ENS remodeling has been reported to change from inflammatory injury and repair to tumor-associated glial and neural reorganization. In colitis models, altered GFAP, C3, and S100A10 expression marks glial-state changes (141). Enteric glia can release CCL2 and CSF1, recruit monocytes, and support CD206+ macrophage states during resolution (142). This adaptive inflammatory response is GI-physiology evidence with preclinical maturity; it could create a permissive environment, but it is not equivalent to a proven precursor of CRC immune escape.

Neural and glial features change across precancerous and invasive CRC. Conventional adenomas and sessile serrated lesions contain fewer Sox10+ enteric glial cells than adjacent mucosa, while sessile serrated lesions can show increased VIP+ neurites and PDK1-RSK-associated mucin expression (143). In invasive CRC, GFAP+ enteric glia reappear within tumor stroma (144). Higher normalized nerve density and PNI are associated with poorer survival, and chemical denervation reduced growth in selected animal models (145, 146). These findings support stage-dependent remodeling but require prospective validation of causality and therapeutic relevance.

In established CRC, soluble tumor factors can induce tumor-supporting enteric glial states, and PGE2-EP4-EGFR signaling can expand stem-like tumor cells (101). Enteric-glial gene signatures correlate with immune infiltration and outcome (100), but causal and predictive validation is needed. Thus, neural involvement changes across IBD, precancerous lesions, invasive CRC, and metastatic disease rather than progressing through a single linear program (27, 147, 148).

### 5.4 Hepatobiliary extension: contextual rather than core evidence

HCC and cholangiocarcinoma are treated as a hepatobiliary extension because the liver and biliary tract do not share the same intrinsic ENS architecture as the stomach or intestine. Direct evidence remains fragmented. In HCC models, nicotine or cholinergic agonists can activate α7nAChR-JAK2-STAT3 and PI3K-AKT signaling (90), whereas ACh-α5 nicotinic receptor signaling can increase BDNF and neural infiltration in intrahepatic cholangiocarcinoma (60). These tumor-cell and neural-growth effects should not be assumed to reproduce enteric neural circuits.

The translational setting is also distinct. Non-selective β-blockers have established use for portal-hypertension-related indications, but retrospective HCC cohorts do not show consistent immune-checkpoint-inhibitor benefit (158, 159). Hepatic vagal effects on metastasis remain supported mainly by preclinical models (171). Underlying cirrhosis, portal hypertension, etiology, and liver-specific immune architecture therefore require hepatobiliary-specific interpretation rather than direct generalization from PDAC, CRC, or GC.

## 6 Temporal and context-dependent dynamics

Neuro-immune interactions change across disease evolution. During colitis and early repair, glial CCL2/CSF1 and cholinergic pathways can restrain tissue damage; in adenomas and sessile serrated lesions, loss of Sox10+ glia can coexist with increased VIP+ neurites; in invasive CRC, glia and nerves reappear within tumor stroma; and in advanced disease, PNI and cumulative nerve injury can support chronic IL-6/type I interferon signaling and dysfunctional CD8+ T-cell states (8, 141–145). These observations argue for stage-specific sampling and intervention windows rather than a static model.

Neural directionality is also context dependent. Muscarinic signaling can suppress pancreatic tumorigenesis and cancer stemness in selected PDAC models (149), and splenic neural signaling can restrain MDSC expansion through trefoil factor 2 (TFF2) (150). In non-gastrointestinal models, sensory-neuron ablation can accelerate breast cancer or melanoma, sensory activation can increase T-cell responses, and local sympathetic α2-adrenergic signaling can restrict pro-tumor myeloid cells (151–155). A recent synthesis frames directionality as a function of tumor kinetics, tissue architecture, neural state, receptor repertoire, and local versus systemic signaling (156). Age is a plausible modifier but remains sparsely tested.

## 7 Evidence and clinical status of candidate neuro-immune interventions

### 7.1 β-adrenergic blockade and drug repurposing

**Figure 5:**
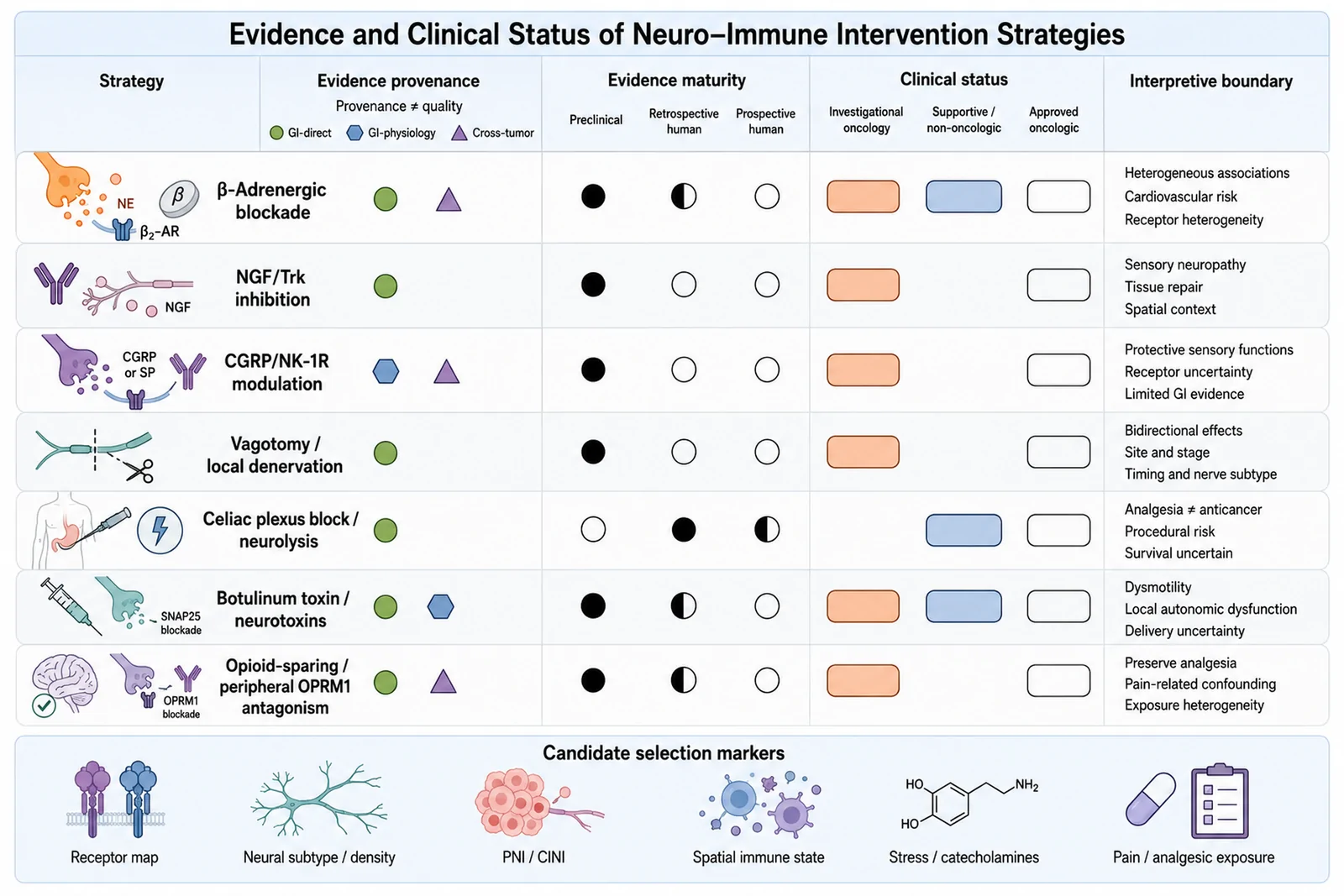
Evidence and clinical status of candidate neuro-immune intervention strategies. Provenance symbols distinguish GI-direct evidence, GI-physiology evidence, and cross-tumor evidence; filled, half-filled, and open circles indicate substantial, limited or mixed, or unestablished evidence at each maturity level. Colored boxes distinguish investigational oncologic from supportive or non-oncologic use; no strategy shown has an approved oncologic indication for neuro-immune modulation. Provenance and maturity/status are descriptive axes, not risk-of-bias grades.

β-adrenergic blockers, particularly propranolol, have preclinical immune-modulating activity, but the clinical evidence is not equivalent across tumor types. **Table 2** and **Figure 5** summarize experimental efficacy, retrospective human associations, established supportive or non-oncologic use, investigational anticancer use, safety concerns, and candidate selection biomarkers; prospective tumor-specific evidence is identified only where available.

**Table 2:** Candidate neuro-immune intervention strategies: evidence provenance, maturity, translational status, limitations, and selection biomarkers.

Strategy	Target/rationale	Provenance, maturity, and translational status	Potential immune outcome	Limitations/safety	Candidate selection biomarkers
β-adrenergic blockade	β-AR-cAMP/PKA signaling in tumor and immune cells	Mixed GI-direct evidence and cross-tumor evidence; preclinical CRC/PDAC evidence and retrospective HCC/PDAC associations; established non-oncologic use in selected settings, but no approved oncologic indication (78–82, 157–159)	May improve effector T-cell activity and reduce suppressive myeloid or angiogenic programs in selected models.	Bradycardia, hypotension, bronchospasm; confounding by cardiovascular or cirrhosis indications; receptor- and tumor-type heterogeneity.	ADRB1/ADRB2 expression by cell type, intratumoral sympathetic density, catecholamine/stress exposure, baseline immune phenotype.
NGF/Trk inhibition	NGF-TrkA/p75NTR-dependent axonal and stromal feedback	GI-direct evidence; preclinical PDAC/CRC evidence; investigational oncologic use with no approved anticancer indication (56, 58, 160–164)	Reduced innervation and neuro-stromal signaling; immune sensitization remains incompletely demonstrated.	Sensory neuropathy and impaired tissue repair; spatially divergent NGFR+ stromal states.	NGF/Trk expression, nerve density, CAF source, PNI, baseline neuropathy and pain.
CGRP/NK-1R pathway modulation	Sensory-neuropeptide signaling to immune and tumor cells	Predominantly cross-tumor evidence or treatment-toxicity evidence; preclinical evidence, with no established gastrointestinal anticancer use (50, 73–76, 92–97)	Potential reduction of suppressive myeloid recruitment or pain-associated inflammation.	Protective sensory-immune functions may be lost; tumor-specific receptor dependence is uncertain.	Sensory-fiber subtype, CGRP/SP abundance, CALCRL/RAMP1 or NK-1R expression, pain phenotype.
Vagotomy or local denervation	Interruption of selected autonomic inputs	GI-direct evidence; preclinical evidence with bidirectional results; no general approved anticancer indication (127, 139, 140, 145, 146, 149, 170, 171)	Can alter T-cell recruitment, myeloid states, metabolism, or metastasis in a model-specific manner.	Irreversible functional loss, dysmotility, metastatic-site effects; timing and completeness are critical.	Nerve subtype and density, tumor site/stage, PNI/CINI score, receptor map, metastatic pattern.
Celiac plexus block/neurolysis	Analgesic interruption of visceral afferent and autonomic pathways	GI-direct evidence; established supportive use for refractory PDAC pain; anticancer or immune benefit remains unproven (165–169)	Reduced pain and opioid requirement; immune outcome is not established.	Hypotension, diarrhea, procedural complications; survival analyses are confounded by disease severity.	Pain severity, opioid exposure, performance status, and disease stage; not an immune-selection biomarker.
Botulinum toxin/neurotoxins	Local blockade of neurotransmitter release, for example SNAP25	GI-direct evidence; established supportive/non-oncologic uses, while anticancer evidence remains preclinical and investigational (139, 172–174)	Potential alteration of tumor metabolism or local neural signaling.	Local autonomic dysfunction, dysmotility, dose and delivery uncertainty.	SNAP25/neural density, anatomical accessibility, motility risk, local versus systemic disease.
Opioid-sparing or peripheral OPRM1 antagonism	Preserve analgesia while reducing peripheral μ-opioid immune signaling	Mixed GI-direct evidence and cross-tumor evidence; *in vitro* or observational GI evidence plus a preclinical non-GI immunotherapy model; prospective GI trials are absent (175–178)	Hypothesized preservation of CD8+ T-cell infiltration and ICI activity without loss of central analgesia.	Pain undertreatment must be avoided; observational confounding and agent/dose heterogeneity.	Cumulative opioid exposure, OPRM1 on immune cells, pain burden, ICI timing, concurrent analgesics.

In CRC models, β2-AR-PKA-SOX10 signaling can increase tumor-cell PD-L1, while β2-AR blockade can improve T-cell infiltration (79). In MC38 tumors, propranolol reduced angiogenesis and MDSCs and improved anti-CTLA-4 activity (80). The active metabolite 4-hydroxypropranolol has also been linked to MAPK inhibition and altered PD-1/Treg readouts in preclinical CRC (157). These observations constitute preclinical, not prospective therapeutic, evidence.

In HCC, non-selective β-blockers are approved for portal-hypertension-related indications, not for immune sensitization (158). Retrospective cohorts of patients receiving ICIs did not show a consistent association between β-blocker use and survival or response (159). Confounding by cirrhosis severity, cardiovascular indication, dose, and β-receptor selectivity limits causal interpretation.

Across gastrointestinal malignancies, clinical associations remain heterogeneous; some meta-analyses in pancreatic cancer report no benefit or possible harm (82). Future trials should stratify by intratumoral nerve density, ADRB2 expression in tumor and immune compartments, baseline catecholamine exposure, tumor immune phenotype, and the clinical indication for β-blockade.

### 7.2 Blockade of neurotrophic signaling

NGF-Trk signaling links axonal growth to stromal and immune remodeling in PDAC and CRC, but direct evidence that pathway inhibition improves ICI efficacy is limited. Nutrient stress can increase PDAC NGF translation and tumor innervation (56), while NGF-TrkA/p75NTR signaling alters nerve, stromal, and immune interactions (160, 161). NGF siRNA delivery and Trk inhibition reduced innervation and tumor growth in preclinical models (56, 162); descriptions of “heating” the tumor microenvironment should be considered hypotheses unless immune and therapeutic endpoints were directly measured.

In CRC, NE-β2-AR signaling in fibroblasts can increase NGF and form a neuro-mesenchymal feedforward loop (58). Adipose-derived NGF/NT-3 and Trk-dependent YAP/AKT pathways also support tumor progression in selected models (163). Conversely, NGFR+ stromal cells can occur in tertiary lymphoid structures associated with favorable outcomes (164). This spatially divergent biology argues for biomarker-guided, local pathway inhibition rather than indiscriminate systemic NGF blockade.

### 7.3 Local denervation, neurolysis, and neurotoxins

Denervation and neurotoxins encompass clinically established analgesic procedures and experimental anticancer interventions; these categories should not be conflated. Their effects depend on the nerve targeted, completeness and timing of denervation, and whether the primary endpoint is pain, tumor growth, or immune function.

Celiac plexus block or neurolysis is used for refractory PDAC pain. Meta-analyses support short-term pain reduction and lower opioid requirements (165, 166), whereas survival effects are uncertain and susceptible to selection bias (167). Endoscopic ultrasound guidance and radiofrequency techniques can improve procedural delivery (168, 169). These approaches are clinically relevant for symptom control but are not approved immune-modulating cancer therapies.

Experimental denervation has produced bidirectional results. Sympathetic or sensory ablation reduced tumor growth in some CRC models (145), whereas subdiaphragmatic vagotomy accelerated PDAC growth in another model (170). In GC and metastatic models, the effect of vagotomy varies between primary-tumor and peritoneal or hepatic metastatic settings (9, 140, 171). These findings argue against tumor-agnostic denervation.

Botulinum toxin has approved non-oncologic and perioperative uses, including chronic anal fissure and management of gastric emptying after esophagectomy (172–174). Intratumoral botulinum toxin A reduced SNAP25-dependent neurotransmission and altered GC metabolism in preclinical work (139). Anticancer use remains investigational; dosing, local autonomic toxicity, gastrointestinal motility, and interactions with standard therapy require prospective evaluation.

### 7.4 Opioid analgesia and neuro-immune consequences

Opioids remain essential for moderate-to-severe cancer pain, particularly in advanced PDAC, and analgesia should not be withheld on the basis of preclinical tumor studies. Nevertheless, opioid effects are receptor-, dose-, timing-, and tissue-dependent. *In vitro* exposure of peripheral blood mononuclear cells from patients with GC increased CD4+/CD8+ ratios and Foxp3+ regulatory T-cell frequencies (175), while morphine produced concentration-dependent, bidirectional effects on pancreatic cancer growth through p38/JNK signaling in cell and xenograft models (176). These studies do not establish harm from clinically indicated analgesia.

Human observational studies linking opioid exposure or μ-opioid receptor expression to shorter survival in PDAC are vulnerable to confounding by pain severity, tumor burden, and performance status (177). In a preclinical oral squamous-cell carcinoma model classified as cross-tumor evidence, morphine reduced CD4+ and CD8+ T-cell infiltration and anti-PD-1 activity through peripheral OPRM1 signaling; a peripherally restricted OPRM1 antagonist restored immune control while preserving central analgesic intent (178). Prospective gastrointestinal studies should record cumulative opioid exposure, agent, route, pain burden, and ICI timing, and should test opioid-sparing or peripheral-antagonist strategies without compromising symptom control.

## 8 Discussion and perspectives

Cancer neuroscience provides a framework for examining a spatial regulatory layer within gastrointestinal tumor immunology. This review integrates anatomy, neural signaling, glial biology, and immunity while separating GI-direct evidence from GI-physiology evidence and cross-tumor evidence. The resulting model is not a single causal circuit but a set of intersecting evidence domains whose contribution varies across PDAC, MSS CRC, GC, HCC, and cholangiocarcinoma (179, 180).

Two recurring mechanisms are anatomically localized remodeling and receptor-specific functional regulation. Neoneurogenesis, PNI, and glial activation can organize stromal and myeloid niches, but evidence for uniform CD8+ T-cell exclusion is incomplete (63, 64). NE-β2-AR signaling can impair T-cell metabolism and function, whereas cholinergic pathways can either support homeostasis or alter tumor and immune states depending on receptor and tissue context (181, 182).

Microbiota-derived metabolites add a gastrointestinal-specific dimension by influencing enteric neurons, glia, epithelial barriers, and immune cells (183, 184). Direct evidence for a complete microbiota-vagus-spleen-tumor circuit remains limited, so microbiota-neural interventions should be evaluated with simultaneous microbial, neural, and immune measurements rather than inferred from any single evidence domain.

Clinical translation of candidate neuro-immune interventions should proceed through evidence-matched trials. β-blockers, anti-NGF/Trk approaches, denervation, and botulinum toxin have distinct clinical statuses and safety profiles; none should be assumed to convert all poorly inflamed tumors into immune-responsive disease (185–189). Biomarker strategies should include neural subtype and density, receptor expression in defined cell populations, PNI or nerve-injury scores, myeloid spatial states, and baseline autonomic or analgesic exposure.

Several limitations qualify this synthesis. The literature is dominated by cell and animal models, uses non-equivalent neural perturbations, and often treats nerve density as a surrogate for function. Human evidence is largely retrospective and vulnerable to confounding by disease stage, pain, treatment indication, cirrhosis, or autonomic comorbidity. GI-physiology evidence and cross-tumor evidence establish plausibility but cannot demonstrate tumor-specific causality. Because this was a narrative review without duplicate screening or a formal study-level risk-of-bias assessment, selection and interpretive bias cannot be excluded. Translational priorities therefore require prospective, tumor-specific validation.

Priority questions include when neural inputs are protective or harmful, how these effects change with age and disease stage, and whether local neuromodulation can preserve physiological innervation while disrupting a tumor-supporting circuit (190, 191). Spatial transcriptomics, multiplex imaging, and functional neural mapping may help identify candidate intervention windows (192). Progress is likely to require tumor-specific prospective studies that integrate immunotherapy, neuromodulation, pain management, and microbiota biology without presuming a universal direction of neural action.
